# Effect of omega-3 supplementation on the nutritional status of patients with pancreatic cancer: A protocol for systematic review of clinical trials

**DOI:** 10.1097/MD.0000000000031262

**Published:** 2022-12-16

**Authors:** Luciana Bicalho Cevolani Pires, Luciane Bresciani Salaroli, Luís Carlos Lopes-Júnior

**Affiliations:** a Graduate Program in Nutrition and Health, Health Sciences Center at the Federal University of Espírito Santo (UFES), Vitória, Brazil.

**Keywords:** fatty acids, nutritional status, omega-3, pancreatic cancer

## Abstract

Globally, pancreatic cancer is the seventh most common cause of cancer-related death in both sexes, accounting for 466,003 deaths and 495,773 new cases in 2020. The purpose of this study was to synthesize and evaluate evidence on the effects of omega-3 supplementation on the nutritional status of patients with pancreatic cancer.

**Methods:**

This systematic review protocol will be guided by Preferred Reporting Items for Systematic Reviews and Meta-Analyses Protocols. Five databases will be searched: MEDLINE/PubMed, CENTRAL Cochrane, EMBASE, Web of Science, and SCOPUS, with no restrictions on the publication date nor language. The internal validity and risk of bias of randomized controlled trials will be assessed using the revised Cochrane Risk-Of-Bias tool for randomized trials (RoB 2), whereas the risk of bias in non-randomized studies of interventions will be evaluated using the ROBINS-I. The heterogeneity among the studies will be assessed using the *I*^2^ statistic. Based on the results of this test, we will verify whether the meta-analysis would be feasible. If feasibility would be confirmed, a random-effect model analysis will be performed. For data analysis, the calculation of the pooled effect estimates will have a 95% confidence interval, while the alpha will be set to 0.05 using the *R* statistical software version 4.0.4. All methodological steps of this review will be performed independently by two reviewers and will be conducted and managed in the EPPI-Reviewer Software™.

**Results:**

This review may be of particular interest to researchers and clinicians, given the low survival and overall burden of patients with pancreatic cancer. Furthermore, the results of this systematic review may contribute to the development of new nutritional interventions in these patients.

**Conclusion:**

To the best of our knowledge, this will be the first study to critically assess the scientific evidence and estimate the effect of omega-3 supplementation on the nutritional status of patients with pancreatic cancer. The review will perform a rigorous approach, adhering to the PRISMA Statement 2020 using a comprehensive and systematic search strategy in five databases and additional sources with no time period nor language restrictions.

## 1. Introduction

Globally, pancreatic cancer is the seventh most common cause of cancer-related death in both sexes, accounting for 466,003 deaths and 495,773 new cases in 2020.^[1]^ Although pancreatic cancer is not among the most prevalent neoplasm, pancreatic cancer has a relatively equivalent incidence and mortality rate.^[1]^ In addition, pancreatic cancer is projected to become the third leading cause of cancer-related death by 2025, as indicated by a study involving 28 European countries.^[2]^

In the United States, pancreatic cancer is the third leading cause of death in men and women. Additionally, mortality from pancreatic cancer has slowly increased in men, from 12.1/100,000 in the 2000s to 12.7/100,000 in 2019; however, it has remained relatively stable in women, ranging from 9.3/100,000 to 9.6/100,000 over the same period.^[3]^

Pancreatic cancer (PC) is a highly lethal disease. In emerging countries, such as Brazil, tumors account for 2% of all neoplasms and represent 4% of deaths.^[4]^ Surgical resection is the only potentially curative treatment option; however, since these tumors present late, only 20% of pancreatic cancer patients are candidates for surgery. In addition, the prognosis is poor, even in patients with a resectable tumor.^[5]^

The 5-year survival rate is approximately 25% to 30%.^[6]^ Advances in survival for pancreatic cancer have been slow. The most important factor is the late stage at which most individuals are diagnosed. Additionally, the late stage 5-year survival rate is 11%.^[3]^ Moreover, the tumor biology is aggressive, and the involvement of other organ systems is likely due to the function and anatomical location of the pancreas.^[7]^ At the same time, most people with pancreatic cancer are asymptomatic until the disease progresses to an advanced stage.^[8]^

Pancreatic cancer has a higher incidence in adults and the elderly. In particular, it is more common after 60 years of age. Approximately 10% of pancreatic cancer cases result from hereditary risk factors; hence, a positive family history of pancreatic cancer substantially increases an individual’s risk of developing the disease.^[8]^ In 2018, the World Cancer Research Fund and the American Institute for Cancer Research concluded that there was strong evidence that being overweight or obese increased the risk of pancreatic cancer.^[9]^ In addition, there is evidence that consumption of red meat, processed meat, foods containing saturated fatty acids, alcoholic beverages, and fructose-containing foods and beverages may increase the risk of pancreatic cancer. Tobacco use is another established cause of pancreatic cancer.^[9,10]^

Weight loss among pancreatic cancer patients is very common, both at the time of diagnosis and during the course of the disease, with up to 40% of patients having a weight loss greater than 10% within 6 months.^[11]^ Several studies showed that malnutrition was associated with the loss of skeletal muscle mass and fat degradation, increased length of hospital stay, increased risk of morbidity and mortality, and reduced treatment response and quality of life.^[7,12,13]^

Cachexia is characterized by pathological weight loss due to loss of skeletal muscle mass and adipose tissue as a result of reduced food intake or anorexia, abnormal metabolism, or a combination of both. Therefore, nutritional interventions play a key role in the successful management of these patients.^[14]^

For the prevention and treatment of cachexia, supplementation with omega-3 polyunsaturated fatty acids, including eicosapentaenoic acid (EPA) and docosahexaenoic acid, has been a strategy in patients with cancer because of the omega-3 polyunsaturated fatty acids’ anti-inflammatory properties. Inflammatory disorders have been managed with EPA and docosahexaenoic acid, which have contributed to the reduction in pro-inflammatory cytokines and maintenance and/or increase in body weight.^[15,16]^

Barber et al^[17]^ provided 20 patients with pancreatic cancer omega-3 polyunsaturated fatty acids (2.2 g EPA/d total). As a result, the patients had stabilized body weight (about 1 kg increase) after 3 weeks of supplementation, along with improvements in terms of appetite and performance status.

Although some systematic reviews explored the modulatory role of omega-3 in patients with head and neck cancer,^[18]^ surgery for gastrointestinal malignancy,^[19]^ prostate cancer,^[20]^ and lung cancer undergoing radiotherapy and chemotherapy,^[21]^ there had been no evidence of its role in the nutritional status of patients with pancreatic cancer.

Thus, to fill this gap in the scientific literature, we hypothesized whether supplementation with omega-3 polyunsaturated fatty acids, applied before or during the treatment of pancreatic cancer, could help prevent malnutrition and muscle wasting in patients with pancreatic cancer. Hence, the aim of this study was to synthesize and evaluate evidence on the effects of omega-3 supplementation on the nutritional status of patients with pancreatic cancer.

## 2. Methods

### 2.1. Study design

This systematic review protocol is in high compliance with the Preferred Reporting Items for Systematic Reviews and Meta-Analyses Protocols.^[22]^ Furthermore, registration was performed using the PROSPERO/UK under registration, CRD42022332619.

### 2.2. Research question

To formulate the research question, the PICOS (P: population or patients; I: intervention; C: comparison; O: outcomes; S: study design) strategy was used.^[23]^ Table 1 shows the acronym, PICOS, used in this protocol for a systematic review.

**Table 1:** Research question elaborated based on PICOS strategy.

PICOS Acronym	Definition	Description
P	Patient/population	Patients [>18 yr old] with pancreatic cancer diagnosis
I	Intervention	Omega-3 supplementation
C	Comparison	Placebo
O	Outcomes	Primary outcome: nutritional status
S	Study design	Randomized Controlled Trials (RCTs) and Non-Randomized Controlled Trials (Quasi-experimental studies)

The PICOS guided the structuring of the research question. The research question is as follows: “What is the effect of omega-3 supplementation on the nutritional status of patients with pancreatic cancer?”

### 2.3. Search strategy

The search strategy will be carried out in five electronic databases: Medical Literature Analysis and Retrieval System Online (MEDLINE) via PubMed [from 1947 to October 31, 2022], Cochrane Central Register of Controlled Trials (from 1991 to October 31, 2022), Excerpta Medica database (from 1973 to October 31, 2022), Web of Science (from 1985 to October 31, 2022), and SCOPUS (from 2004 to October 31, 2022). The strategy for seeking studies will consist of a combination of controlled descriptors (indexers in the respective databases, e.g., Medical Subject Headings [MeSH] terms, Emtree terms), synonyms, and keywords, as indicated in each electronic database.^[24]^ The Boolean operators “AND,” “OR,” and “NOT” will be employed to combine the descriptors.^[25,26]^ No restrictions regarding language nor period of publication will be applied in the search strategy. In addition to the electronic databases cited above, searches will be conducted on clinical trial registry sites, such as ClinicalTrials. gov (National Institutes of Health, NIH, USA), World Health Organization International Clinical Trials Registry Platform, and additional searches in sites of organizations and website searches, such as the British Library, Google Scholar, and preprints for Health Sciences [medRxiv]. In addition, we will scrutinize the reference lists of articles to search for additional studies.^[27]^

In this phase, the EndNote bibliographic software will be used to store, organize, and manage all retrieved studies. The search strategy will be conducted independently by two researchers (LCLJ and LBCP). First, the existence of an index of specific subject headings in each database (such as MeSH terms, Emtree terms, and their synonyms and keywords) will be identified. Subsequently, the search terms will be combined using the Boolean operators “AND” and “OR”^[24–27]^ to obtain restrictive and additive combinations, respectively. In addition, the search will be performed using the identified descriptors with their expanded meaning but without the use of database filters to preserve significant samples and to ensure a lower risk of loss. The search strategy combining the MeSH controlled descriptors and keywords used in MEDLINE will be adjusted to other electronic databases, as described in Table 2.

**Table 2:** Preliminary search strategy in the MEDLINE via PubMed.

Database	Items searched
MEDLINE/PubMed	#1 (Adult[mesh terms] OR Adults[tiab] OR Young Adult[mesh terms] OR Adult, Young[tiab] OR Adults, Young[tiab] OR Young Adults[tiab] OR Middle Aged[mesh terms] OR Middle Age[tiab] OR “Aged”[mesh terms] OR Elderly[tiab]OR Aged, 80 and over[mesh terms] OR Oldest Old[tiab])
	#2 (Pancreatic Neoplasms[mesh terms] OR Neoplasm, Pancreatic[tiab] OR Pancreatic Neoplasm[tiab] OR Pancreas Neoplasms[tiab] OR Neoplasm, Pancreas[tiab] OR Neoplasms, Pancreas [tiab] OR Pancreas Neoplasm[tiab] OR Neoplasms, Pancreatic[tiab] OR Cancer of Pancreas[tiab] OR Pancreas Cancers[tiab] OR Pancreas Cancer[tiab] OR Cancer, Pancreas[tiab] OR Cancers, Pancreas[tiab] OR Pancreatic Cancer[tiab] OR Cancer, Pancreatic[tiab] OR Cancers, Pancreatic[tiab] OR Pancreatic Cancers[tiab] OR Cancer of the Pancreas[tiab] OR Pancreatic cancer, adult[tiab])
	#3 #1 AND #2
	#4 (Fatty Acids, Omega-3[mesh terms] OR Omega-3 Fatty Acid[tiab] OR Acid, Omega-3 Fatty[tiab] OR Fatty Acid, Omega-3[tiab] OR Omega 3 Fatty Acid[tiab] OR Omega-3 Fatty Acids[tiab] OR n-3 Oil[tiab] OR Oil, n-3[tiab] OR n 3 Oil[tiab] OR Oil, n3[tiab] OR n-3 Fatty Acids[tiab] OR n 3 Fatty Acids[tiab}OR Omega 3 Fatty Acids[tiab] OR n-3 PUFA[tiab] OR PUFA, n-3[tiab] OR n 3 PUFA[tiab] OR n3 Fatty Acid[tiab] OR Fatty Acid, n3[tiab] OR n3 PUFA[tiab] OR PUFA, n3[tiab] OR n3 Polyunsaturated Fatty Acid[tiab] OR n-3 Oils[tiab] OR n 3 Oils[tiab] OR N-3 Fatty Acid[tiab] OR Acid, N-3 Fatty[tiab] OR Fatty Acid, N-3[tiab] OR N 3 Fatty Acid[tiab] OR n-3 Polyunsaturated Fatty Acid[tiab] OR n 3 Polyunsaturated Fatty Acid[tiab] OR Eicosapentaenoic Acid[mesh terms] OR 5,8,11,14,17-Eicosapentaenoic Acid[tiab] OR Eicosapentanoic Acid[tiab] OR Acid, Eicosapentanoic[tiab] OR omega-3-Eicosapentaenoic Acid[tiab] OR omega 3 Eicosapentaenoic Acid[tiab] OR Timnodonic Acid[tiab] OR Icosapent[tiab] OR 5,8,11,14,17-Icosapentaenoic Acid[tiab] OR Docosahexaenoic Acids[mesh terms] OR Acids, Docosahexaenoic[tiab}OR Docosahexenoic Acids[tiab] OR Acids, Docosahexenoic[tiab] OR Docosahexaenoic Acid[tiab] OR Acid, Docosahexaenoic[tiab]OR Docosahexaenoic Acid[tiab] OR Acid, Docosahexaenoic[tiab] OR Docosahexaenoate[tiab] OR alpha-Linolenic Acid[mesh terms] OR Linolenic Acid[tiab])
	#5 #3 AND 4
	#6 (Nutritional Status[mesh terms] OR Status, Nutritional[tiab] OR Nutrition Status[tiab] OR Status, Nutrition[tiab])
	#7 #5 AND #6
	#8 (randomized controlled trial[pt] OR controlled clinical trial[pt] OR randomized[tiab] OR placebo[tiab] OR clinical trials as topic[mesh:noexp] OR randomly[tiab] OR trial[ti] NOT (animals[mh] NOT humans[mh]))
	#9 #7 AND #8

MEDLINE = Medical Literature Analysis and Retrieval System Online.

### 2.4. Eligibility

Inclusion criteria: All primary studies from randomized clinical trials and non-R randomized clinical trials, which assessed the effect of omega-3 supplementation in adult patients (>18 years old) of both sexes and of any ethnicity with a diagnosis of pancreatic cancer. This will include clinical trials with the nutritional status as an outcome. Notably, no date or language restrictions will be applied during study selection.

• Exclusion criteria: Experimental studies conducted in animal models, in vivo, and ex vivo on this topic will be excluded. Observational studies, qualitative studies, reviews, and gray literature will also be excluded.

Studies will be selected by the same two reviewers independently and blindly. After this selection, a third reviewer will be responsible for analyzing and deciding on the inclusion or exclusion of each article, especially those containing conflicting decisions.^[22]^ In this step of inclusion and exclusion of articles that will compose the final sample, the Rayyan™-Qatar CRI^[28]^ will be used as a tool to assist in the eligibility and selection of articles.

### 2.5. Data extraction

First, the screening of the studies will be based on the information contained in their titles and abstracts and will be performed by the same two researchers (LCLJ and LBCP) using previously published data extraction forms.^[27,29–37]^ If the reviewers disagree, the article will be evaluated. If disagreement will persist, a third reviewer will make the final decision.

The information to be extracted^[32]^ is detailed in Table 3.

**Table 3:** The data extraction form was elaborated based on previous publications.^[27,29–37]^

Item	Data to be extracted
Study characteristics	Authors
	Title
	Year of publication
	Country
	Conflicts of interests
	Sponsorship
	Background
	Rationale
	Hypothesis
	Objectives
	Methodology is reported is in compliance with CONSORT (clinical trials)
	() Yes () No () Partially
	Study design
	Trial Register:
	Trial arms:
	• Experimental Group
	• Control Group
	Local:
	Sample size:
	Randomization:
	Masking:
	Inclusion criteria (definition of exposure of interest)
	Exclusion criteria
Methods	Confounding factors/Interaction factors considered
	Ethical aspects
	Procedure for data collection:
	Instruments for data collection
	Outcomes
	Primary outcome
	Secondary outcome
	Intervention protocol:
	Per-protocol and modified intention-to-treat analyses:
	-Per-protocol:
	-Intention-to-treat:
	-Dropouts:
	Follow-up
	Statistical analysis
	Main results
Results	Clinical significance
	Limitations of the study
	Strengths of the study
	Main conclusions
Conclusions	Implication for clinical practice and research or for decision-makers/stakeholders

### 2.6. Methodological appraisal

The internal validity and risk of bias of randomized controlled trials will be assessed using the revised Cochrane Risk-Of-Bias tool for randomized trials (RoB 2).^[38]^ This tool will be used to assess the risk of bias in five domains: randomization process, deviations from the intended interventions, missing outcome data, outcome measurement, and selection of the reported result. The RoB 2 will be used to classify the risk of bias as follows: low risk of bias: low risk of bias for all domains; some concerns: some concerns in at least one domain, but no high risk of bias for any domain; and high risk of bias: high risk of bias in at least one domain or some concerns for multiple domains, substantially reducing confidence in the results. To assess non-R randomized controlled trials, the risk of bias in non-randomized studies of interventions (ROBINS-I)^[39]^ will be used. ROBINS-I will comprise of seven chronologically arranged bias domains (pre-intervention, at intervention, and post-intervention). The domain level and overall risk of bias will be classified as low, moderate, serious, or critical. For both tools, the same two reviewers will independently assess the risk of bias for each included study. Discrepancies will be resolved through discussion at each stage until the achievement of a consensus.

### 2.7. Data analysis and evidence synthesis

Heterogeneity among studies will be measured using the *I*^2^ statistic to estimate the percentage of variation among studies, where *I*^2^ = 0–40% will indicate low heterogeneity; *I*^2^ = 30–60% will indicate moderate heterogeneity; *I*^2^ = 50–90% will indicate substantial heterogeneity; and *I*^2^ = 75–100% will indicate high heterogeneity.^[40,41]^ Based on the *I*^2^ statistic, we will determine whether the meta-analysis would be feasible. In this case, we will check the statistical model to be used to gather the study-specific estimates, which will be in the form of fixed-effects model or random-effects model analysis.^[32–34,42–44]^ For the data analysis, the calculation of the pooled effect estimates will consider a confidence interval (CI) of 95% and an α = 0.05 using the EPPI-Reviewer™ Software (UK). We will also assess the publication bias if sufficient studies would be identified per endpoint analyzed.^[45]^ To explore the heterogeneity across studies, a subgroup analysis will be performed using a mixed effects model according to age (young people versus adults versus aged versus added patients) and cancer staging. For the data analysis, the calculation of the pooled effect estimates will consider a 95% CI, while α alpha will be set at 0.05 using the Statistical Package for Social Sciences version 28.0 (SPSS® Inc., Chicago, IL).

**Figure 1.**
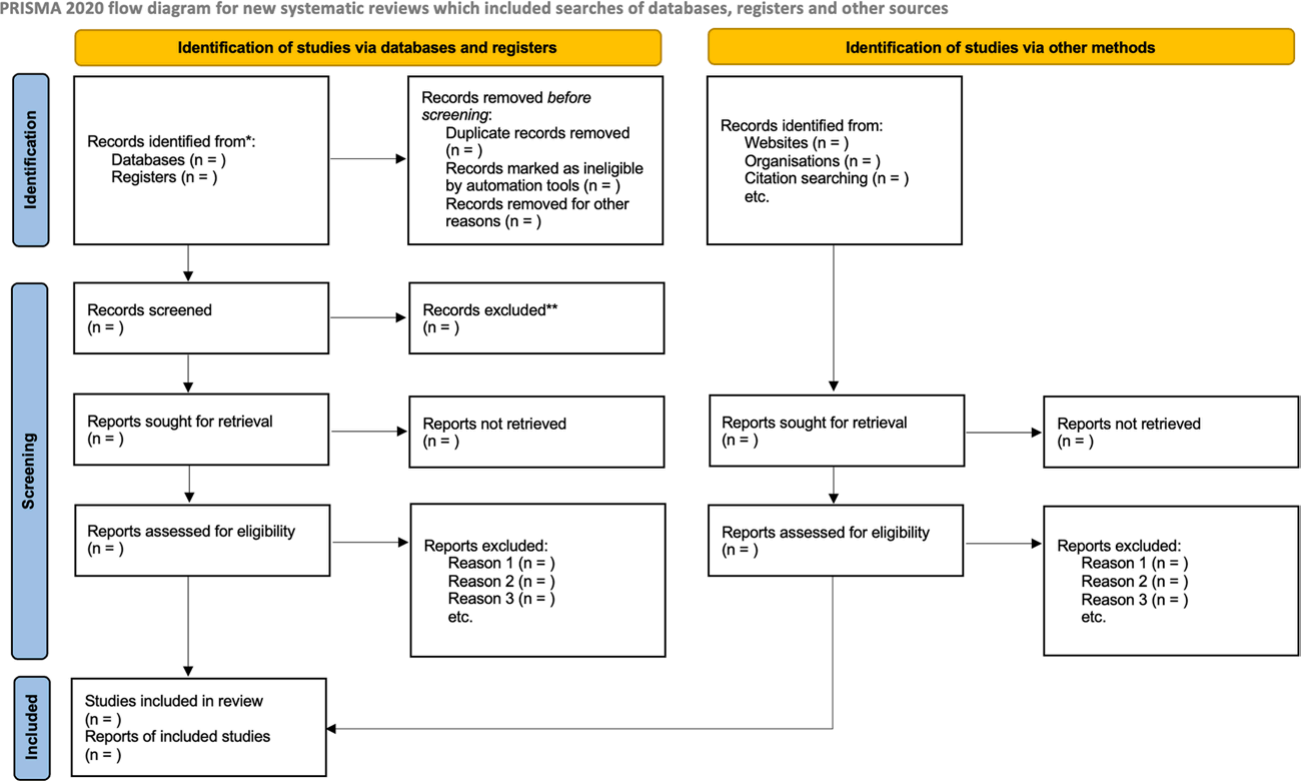
PRISMA flow diagram. PRISMA = Preferred Reporting Items for Systematic Reviews and Meta-Analyses Protocols. *Consider, to reporting the number of records identified from each database or register searched (rather than the total number across all databases/registers). **If automation tools were used, indicate how many records were excluded by a human and how many were excluded by automation tools.

The study results will be presented in the Preferred Reporting Items for Systematic Reviews and Meta-Analyses 2020^[46]^ flowchart (Fig. 1), tables, or graphs in the same way the summaries would be reported.^[24]^

### 2.8. Patient and public involvement

This protocol for systematic review will analyze the existing research studies and therefore will not involve patients nor members of the public.

### 2.9. Ethics and dissemination

This study will not involve human participants or unpublished primary data. As such, ethical approval from the Human Research Ethics Committee will not be required. Plans for the dissemination of this study will include peer-reviewed publications and conference presentations to be shared with experts in this field, stakeholders and clinicians.

## 3. Discussion

This systematic review will help in the elucidation of the current state of knowledge and will identify gaps in the literature on nutritional status and omega-3 interventions in patients with pancreatic cancer. In addition, this review may be of particular interest to researchers and clinicians, given the low survival and overall burden of patients with pancreatic cancer. Moreover, the results of this systematic review may contribute to the development of new nutritional interventions in these patients.

To the best of our knowledge, this systematic review will be the first to critically assess scientific evidence and to estimate the effect of omega-3 supplementation on the nutritional status of patients with pancreatic cancer. Thus, we will provide better insights into omega-3 supplementation in patients with pancreatic cancer, a disease with a high fatality and mortality rate in both sexes. Finally, this may provide consistent evidence that will assist decision makers and stakeholders.

## Author contributions

LCLJ and LBCP conceived the idea and planned and designed the study protocol. LCLJ and LBCP wrote the first draft. LCLJ planned the data extraction and statistical analysis. LCLJ, LBCP and LBS provided critical insights. LCLJ and LBCP critically reviewed and modified the manuscript. All authors have reviewed and approved the manuscript. LCLJ is responsible for the overall content as guarantor.

**Conceptualization:** Luciana Bicalho Cevolani Pires, and Luís Carlos Lopes-Júnior.

**Data curation:** Luciana Bicalho Cevolani Pires, Luciane Bresciani Salaroli, Luís Carlos Lopes-Júnior.

**Formal analysis:** Luís Carlos Lopes-Júnior.

**Funding acquisition:** Luís Carlos Lopes-Júnior.

**Investigation:** Luís Carlos Lopes-Júnior.

**Methodology:** Luís Carlos Lopes-Júnior.

**Project administration:** Luís Carlos Lopes-Júnior.

**Resources:** Luís Carlos Lopes-Júnior.

**Software:** Luís Carlos Lopes-Júnior.

**Supervision:** Luís Carlos Lopes-Júnior.

**Validation:** Luciane Bresciani Salaroli, Luís Carlos Lopes-Júnior.

**Visualization:** Luciana Bicalho Cevolani Pires, Luciane Bresciani Salaroli, Luís Carlos Lopes-Júnior.

**Writing – original draft:** Luciana Bicalho Cevolani Pires, Luciane Bresciani Salaroli, Luís Carlos Lopes-Júnior.

**Writing – review & editing:** Luciana Bicalho Cevolani Pires, Luís Carlos Lopes-Júnior.
